# Tangeretin Ameliorates Glucose-Induced Podocyte Injury through Blocking Epithelial to Mesenchymal Transition Caused by Oxidative Stress and Hypoxia

**DOI:** 10.3390/ijms21228577

**Published:** 2020-11-13

**Authors:** Min-Kyung Kang, Soo-Il Kim, Su Yeon Oh, Woojin Na, Young-Hee Kang

**Affiliations:** Department of Food science and Nutrition and The Korean Institute of Nutrition, Hallym University, Chuncheon 24252, Korea; ky4850@naver.com (S.-I.K.); suy0411@naver.com (S.Y.O.); nsm0729@hanmail.net (W.N.)

**Keywords:** diabetic nephropathy, glucose, hypoxia, podocyte injury, epithelial to mesenchymal transition

## Abstract

Podocyte injury inevitably results in leakage of proteins from the glomerular filter and is vital in the pathogenesis of diabetic nephropathy (DN). The underlying mechanisms of podocyte injury facilitate finding of new therapeutic targets for DN treatment and prevention. Tangeretin is an O-polymethoxylated flavone present in citrus peels with anti-inflammatory and antioxidant properties. This study investigated the renoprotective effects of tangeretin on epithelial-to-mesenchymal transition-mediated podocyte injury and fibrosis through oxidative stress and hypoxia caused by hyperglycemia. Mouse podocytes were incubated in media containing 33 mM glucose in the absence and presence of 1–20 μM tangeretin for up to 6 days. The in vivo animal model employed db/db mice orally administrated with 10 mg/kg tangeretin for 8 weeks. Non-toxic tangeretin inhibited glucose-induced expression of the mesenchymal markers of N-cadherin and α-smooth muscle actin in podocytes. However, the reduced induction of the epithelial markers of E-cadherin and P-cadherin was restored by tangeretin in diabetic podocytes. Further, tangeretin enhanced the expression of the podocyte slit diaphragm proteins of nephrin and podocin down-regulated by glucose stimulation. The transmission electron microscopic images revealed that foot process effacement and loss of podocytes occurred in diabetic mouse glomeruli. However, oral administration of 10 mg/kg tangeretin reduced urine albumin excretion and improved foot process effacement of diabetic podocytes through inhibiting loss of slit junction and adherenes junction proteins. Glucose enhanced ROS production and HIF-1α induction in podocytes, leading to induction of oxidative stress and hypoxia. Similarly, in diabetic glomeruli reactive oxygen species (ROS) production and HIF-1α induction were observed. Furthermore, hypoxia-evoking cobalt chloride induced epithelial-to-mesenchymal transition (EMT) process and loss of slit diaphragm proteins and junction proteins in podocytes, which was inhibited by treating submicromolar tangeretin. Collectively, these results demonstrate that tangeretin inhibited podocyte injury and fibrosis through blocking podocyte EMT caused by glucose-induced oxidative stress and hypoxia.

## 1. Introduction

Chronic hyperglycemia causes loss of specific proteins of podocytes, leading to glomerulopathy and renal failure [1]. Podocytes along with their specialized junctions of slit diaphragm are involved in maintaining the permeability of the glomerular filtration barrier [2,3]. The slit diaphragm is the intercellular junction between interdigitating foot processes of podocytes and is composed of diverse proteins such as podocin, nephrin, CD2-associated protein, Zonula occludens-1 (ZO-1) and α-actinin 4 [4]. The integrity of the slit diaphragm is critical for maintaining the actin cytoskeleton and the function of the glomerular filtration assembly. Severe injury of the slit diaphragm complex lead to loss or effacement of podocyte foot process and, which contributes to protein uric renal injury [4,5]. In addition, the disruption of the filtration barrier observed in diabetic nephrotic diseases, is due to damage of proteins within the slit diaphragm such as nephrin [6]. Accordingly, the podocytes can be a potential therapeutic target in diabetic nephropathy (DN). Some currently used therapies, such as antioxidants, statins and inhibitors of glycation inhibit podocyte loss [7].

Epithelial-to-mesenchymal transition (EMT) is a tightly regulated process by which epithelial cells lose their epithelial characteristics and acquire the mesenchymal features [8,9]. During the podocyte EMT the expression of nephrin, podocin and ZO-1 is downregulated, the slit diaphragm is altered and the actin cytoskeleton is rearranged [10]. On the contrary, podocytes express mesenchymal markers such as desmin, fibroblast-specific protein (FSP)-1 and matrix metalloproteinase proteins during EMT process [10]. The podocyte EMT process is known to occur in diabetic kidney diseases [9,10]. Hyperglycemia induces podocyte EMT through several molecular mechanisms such as TGF-β/Smad classic pathway, integrin-linked kinase signaling pathway and NF-κB signaling pathway [10,11]. In order to develop therapeutic options targeting diabetic kidney disease, an understanding of these molecular mechanisms responsible for the pathogenesis of podocyte EMT is compulsory.

Hypoxia is a potent regulator of multiple cellular processes including metabolism, growth and cell survival via the mechanisms of oxygen sensing [12]. The transcriptional responses to oxygen deprivation are mainly mediated by hypoxia-inducible factors (HIF), which operates during normal development and in pathological processes in association with decreased oxygen availability [13]. The kidney is prone to hypoxic injury owing to an arterio-venous oxygen shunt [14]. Hyperglycemia increases oxygen consumption in mitochondria, consequently resulting in cellular hypoxia and generation of reactive oxygen species (ROS) [15]. A large body of evidence indicates that diabetic glucose loading induces chronic hypoxia that is one of the common pathogenic mechanisms driving the progression of DN [16,17]. A study reports that the activation of HIF signaling in renal epithelial cells may promote fibrogenesis by facilitating EMT [18]. Alteration in oxygen levels and hypoxic signaling activation through HIF are emerging as important triggers and modulators of EMT [19]. However, the precise mechanisms underlying hypoxia-induced nephrotic injury remain unclear.

Several potential drugs, as well as biologicals, have been identified for proteinuria and kidney diseases through direct podocyte targeting [20]. In addition, naturally-occurring polyphenols protects against podocyte injury and proteinuria via diverse mechanisms [21,22,23]. Tangeretin is an O-polymethoxylated flavone that is found in tangerine and various citrus peels. Tangeretin has diverse pharmacological activities and possesses multiple biological effects. Several reports have shown that tangeretin exhibits antioxidant, neuroprotective effects, anti-apoptosis and anti-cancer effects [24,25,26]. However, little is known about its renoprotective effects. This study investigated hyperglycemia-mediated hypoxia and oxidative stress induced EMT-triggered fibrotic injury of podocytes, which was inhibited by treating tangeretin to diabetic podocytes. In addition, the current study employed 33 mM glucose and cobalt chloride (CoCl_2_) in order to induce oxidative stress and hypoxia in podocytes. CoCl_2_ is shown to artificially induce hypoxia and accumulates HIF-1α protein as a hypoxia-mimetic agent through activating ERK1/2 and PI3K signaling pathways [27,28].

## 2. Results

### 2.1. Inhibitory Effects of Tangeratein on Glucose-Induced Podocyte EMT

**Figure 1:**
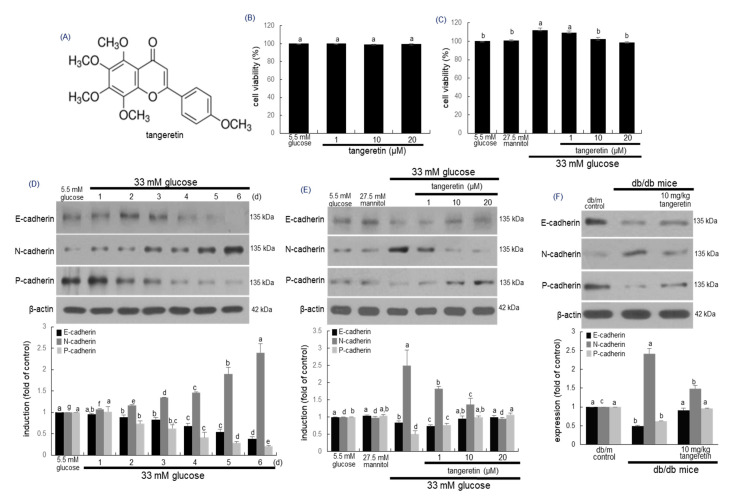
Chemical structure (**A**) and cytotoxicity (**B**,**C**) of tangeretin, temporal expression of E-cadherin, N-cadherin and P-cadherin in podocytes (**D**) and modulation of their induction by tangeretin in podocytes and diabetic kidney (E and F). Podocytes were incubated with 33 mM glucose in the presence of 1–20 μM tangeretin for 4 days. Cell viability (mean ± SEM, *n* = 5) was measured by MTT assay and expressed as percent cell survival relative to glucose controls (**B**,**C**). The db/db mice were orally supplemented with 10 mg/kg tangeretin for 10 weeks (**F**). Podocyte lysates and kidney tissue extracts were electrophoresed on 8–10% SDS-PAGE and subject to Western blot analysis with a primary antibody against E-cadherin, N-cadherin and P-cadherin (**D**–**F**). β-Actin antibody was used as an internal control. The bar graphs (mean ± SEM, *n* = 3) in the bottom panels represent quantitative results of upper blot bands obtained from a densitometer. Means in bar graphs without a common letter differ, *p* < 0.05.

The podocytes are highly differentiated epithelial cells responsible for supporting the glomerular tuft and maintaining the structure and function of the glomerular filtration barrier [3]. This study investigated that tangeretin ameliorated the EMT in glucose-loaded podocytes. When podocytes were treated with 1–20 μM tangeretin for 4 day in 33 mM glucose-containing media, tangeretin did not show cytotoxicity, as evidenced by MTT assay (Figure 1B,C). Exposure of podocytes to 33 mM glucose for 6 days, the cellular levels of epithelial markers of E-cadherin and P-cadherin reduced in a temporal manner (Figure 1D). In contrast, the expression of the mesenchymal protein N-cadherin was gradually enhanced (Figure 1D). The induction of E-cadherin, P-cadherin and N-cadherin was reciprocally reversed by treating the podocytes with ≥10 μM tangeretin (Figure 1E). Oral administration of 10 mg/kg tangeretin attenuated the expression of kidney tissue N-cadherin elevated in db/db mice (Figure 1F). However, the expression levels of E-cadherin and P-cadherin were elevated (Figure 1F).

### 2.2. Inhibition of Formation of Myofibroblasts by Tangeretin

**Figure 2:**
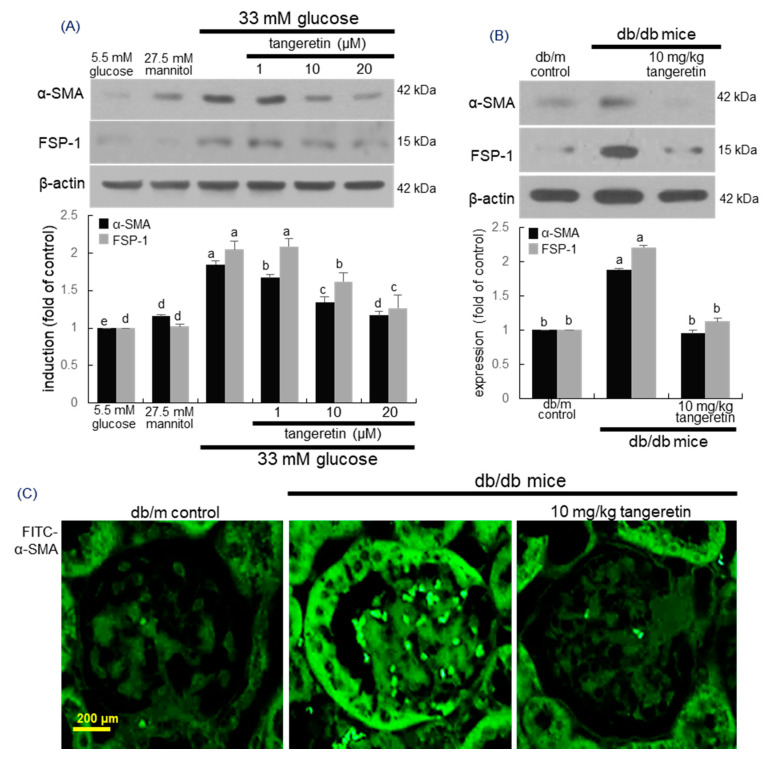
Inhibition of expression of α-SMA and FSP-1 by tangeretin in glucose-exposed podocytes (**A**) and diabetic kidney (**C**). Podocytes were incubated in media containing 5.5 mM glucose, 5.5 mM glucose plus 27.5 mM mannitol as osmotic controls or 33 mM glucose in the absence and presence of 1–20 μM tangeretin for 4 days (**A**). The db/db mice were orally supplemented with 10 mg/kg tangeretin for 10 weeks (**B**,**C**). Cell lysates and kidney extracts were electrophoresed on 10–15% SDS-PAGE and subject to Western blot analysis with a primary antibody against α-SMA and FSP-1 (**A**,**C**). β-Actin antibody was used as an internal control. The bar graphs (mean ± SEM, *n* = 3) represent quantitative results of blot bands obtained from a densitometer. Respective values not sharing a letter are different at *p* < 0.05. Immunohistochemical staining showing glomerular tissue levels of α-SMA of diabetic mouse kidney (**C**). The α-SMA of mouse kidneys was confirmed by FITC-green staining. Each photograph is representative of four mice. Scale bar = 200 μm.

Fibrosis of the kidney glomerulus and interstitium involves the accumulation of fibroblasts with the phenotypic appearance of myofibroblasts, which is one of characteristic features of almost all chronic kidney diseases [29]. The expression levels of the myofibroblast markers of FSP-1 and α-smooth muscle actin (α-SMA) markedly elevated by high glucose were dose-dependently attenuated by 1–20 μM tangeretin (Figure 2A). In addition, oral administration of 10 mg/kg tangeretin reduced the kidney tissue levels of FSP-1 and α-SMA in db/db mice (Figure 2B). The immunohistochemical Fluorescein isothiocyanate (FITC)-staining of α-SMA confirmed that the myofibroblast accumulation in glomeruli was apparent and such accumulation was diminished by oral supplementation of tangeretin to diabetic mice (Figure 2C). Thus, tangeretin may encumber renal fibrosis through inhibiting podocyte EMT process.

### 2.3. Inhibition of Loss of Slit Diaphragm Proteins by Tangeretin

**Figure 3:**
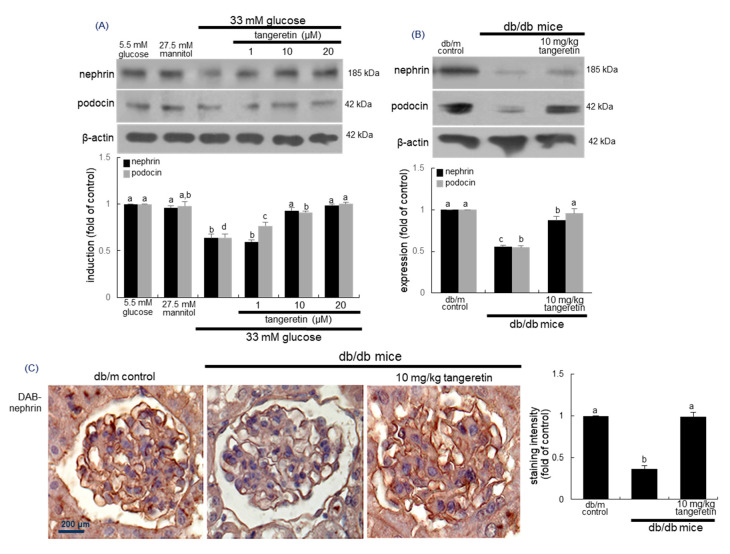
Elevation of induction of nephrin and podocin by tangeretin in podocytes and diabetic kidneys. Podocytes were incubated with 33 mM glucose in the presence of 1–20 μM tangeretin for 4 days (**A**). The db/db mice were orally supplemented with 10 mg/kg tangeretin for 10 weeks (**B**,**C**). Cell lysates and tissue extracts were electrophoresed on 8–12% SDS-PAGE and subject to Western blot analysis with a primary antibody against nephrin and podocin (**A**,**B**). β-Actin antibody was used as an internal control. The bar graphs (mean ± SEM, *n* = 3) in the bottom panels represent quantitative results of upper blot bands obtained from a densitometer. Respective values not sharing a letter are different at *p* < 0.05. Immunohistochemical staining showing glomerular tissue level of nephrin of diabetic mouse kidney (**C**). The glomerular nephrin was confirmed with 3,3′-diaminobenzidine (DAB), producing a brown staining, counterstained with hematoxylin and was quantified. Each photograph is representative of four mice. Scale bar = 200 μm.

Diabetes-associated podocyte injury may cause loss of slit diaphragm proteins of podocytes, detachment from the glomerular basement membrane and proteinuria [30]. This study examined whether the treatment of tangeretin inhibited loss of slit diaphragm proteins in glucose-stimulated podocytes. In podocytes exposed to 33 mM glucose for 4 days, the expression levels of nephrin, podocin and P-cadherin declined (Figure 3A). In contrast, the addition of 1–20 μM tangeretin to diabetic cells dose-dependently enhanced the expression of these proteins.

This study further investigated that oral administration of 10 mg/kg tangeretin inhibited loss of the slit diaphragm proteins in diabetic kidneys. Western blot data revealed that renal tissue levels of nephrin and podocin were reduced in db/db mice (Figure 3B). When 10 mg/kg tangeretin was orally administrated to db/db animals for 10 weeks, the induction of these proteins increased in diabetic mouse kidneys. Consistent with the Western blot data (Figure 3B), the tangeretin supplementation ameliorated the reduction of glomerular nephrin level in diabetic mice, as evidenced by immunohistochemical staining of nephrin (Figure 3C).

### 2.4. Blockade of Podocyte Injury by Tangeretin

**Figure 4:**
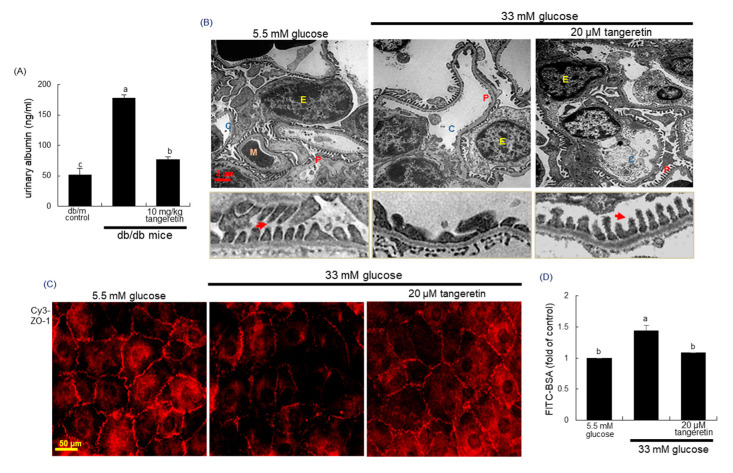
Effects of tangeretin on mouse urinary albumin excretion (**A**) and structural changes in diabetic kidneys (**B**). The db/db mice were supplemented with 10 mg/kg tangeretin for 10 weeks (**A**,**B**). Urinary albumin levels were measured by using ELISA (**A**). Foot process of renal podocytes and ultrastructure of glomeruli were observed in db/m control and db/db mice, evidenced by transmission electron microscopy (TEM) analysis of (**B**). Abbreviations: P, podocytes; M, mesangial cells; E, erythrocytes; C, capillary tuft. Podocytes were incubated with 33 mM glucose in the presence of 20 μM tangeretin for 4 days. The fixed cells were incubated with a primary antibody against ZO-1 and the ZO-1 localization was visualized with a Cy3-conjugated secondary antibody (**C**). Each photograph is representative of stained cells. Scale bar = 3 μm or 50 μm. For the measurement of podocyte injury, albumin permeability assay was employed with FITC-labeled bovine serum albumin (BSA), (**D**). Values (mean ± SEM, *n* = 5) in bar graphs not sharing a common letter are significantly different at *p* < 0.05.

Pathologic alterations and depletion of podocytes contribute the failure of glomerular filtration barrier and proteinuria in diabetic kidneys [8,9]. As expected, there was a severe albuminuria observed in db/db animals but the 10 week-oral supplementation of tangeretin significantly reduced urinary albumin excretion in diabetic animals (Figure 4A). To examine the pathological ultrastructure of the glomeruli in diabetic mice, the transmission electron microscopy (TEM) analysis was conducted. Under TEM, the glomerular morphology was apparently altered in the diabetic mice, compared with that of the non-diabetic animals (Figure 4B). In addition, loss and foot process effacement of podocytes were observed in glomeruli of diabetic mice (yellow arrows). However, such pathological alterations in glomerular ultrastructure were normalized after the oral supplementation of 10 mg/kg tangeretin (Figure 4B).

There are two types of cellular junctions of slit diaphragm and tight junction in the foot process [31]. Immunocytochemical analysis revealed that there was strong Cy3-staining of ZO-1 in 5.5 mM glucose controls (Figure 4C). However, the staining was highly attenuated in glucose-loaded podocytes, indicating that glucose attenuated the induction of the tight junction protein ZO-1 of podocytes (Figure 4C). In contrast, tangeretin reversed the reduced induction of ZO-1 in glucose-exposed podocytes. To confirm the loss of tight junction proteins in diabetic podocytes, the FITC-labeled albumin permeability assay was conducted. In podocytes exposed to 33 mM glucose the FITC fluorescence was elevated, indicating the apparent permeability of BSA (Figure 4D). When 20 μM tangeretin were treated to diabetic podocytes, the albumin permeability was abolished. Thus, tangeretin may encumber podocyte injury and foot process effacement leading to proteinuria in diabetic mice.

### 2.5. Effects of Tangeretin on Glucose-Induced Oxidative Stress in Podocytes

**Figure 5:**
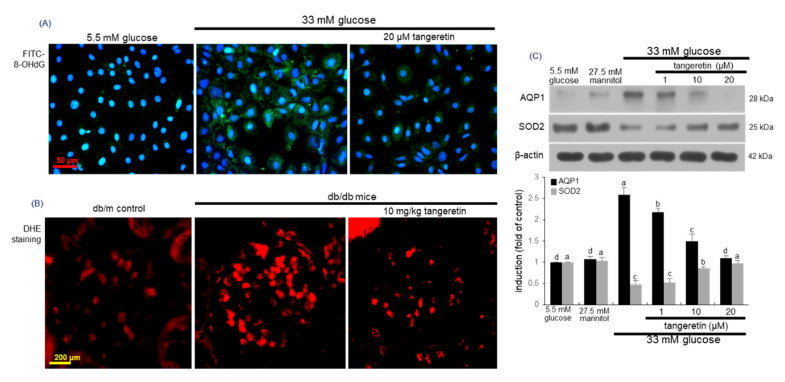
Blockade of 8-OHdG induction (**A**), reactive oxygen species (ROS) production (**B**) and AQP-1 induction (**C**) and elevation of SOD2 induction (**C**) by tangeretin in glucose-exposed podocytes and in diabetic kidneys. Podocytes were incubated in media containing 5.5 mM glucose, 5.5 mM glucose plus 27.5 mM mannitol as osmotic controls or 33 mM glucose in the absence and presence of 1–20 μM tangeretin for 4 days. For the immunocytochemical analysis of 8-OHdG (**A**), a FITC-conjugated secondary antibody was used to visualize the induction of 8-OHdG. The nuclear staining was done with DAPI (blue). Each photograph is representative of stained cells. Magnification: 400-fold. The db/db mice were orally supplemented with 10 mg/kg tangeretin for 10 weeks. For the measurement of ROS generation in kidney glomeruli, the dihydroethidium (DHE) staining was performed (**B**). Each photograph is representative of four mice. Scale bar = 50 μm or 200 μm. Cell lysates and tissue extracts were electrophoresed on 12–15% SDS-PAGE and subject to Western blot analysis with a primary antibody against AQP-1 and SOD2 (**C**). β-Actin antibody was used as an internal control. The bar graphs (mean ± SEM, *n* = 3) represent quantitative results of blot bands obtained from a densitometer. Values in bar graphs not sharing a common letter are significantly different at *p* < 0.05.

Hyperglycemia induces cellular hypoxia and mitochondrial ROS generation, which may promote hyperglycemic damage [15]. This study examined whether glucose loading created oxidative stress in podocytes, which was attenuated by tangeretin. High glucose highly induced oxidative stress, as evidenced by immunocytochemical FITC-green staining of 8-hydroxy-2-deoxy guanosine (8-OHdG), a biomarker of endogenous oxidative DNA damage [32] (Figure 5A). However, the 8-OHdG expression was attenuated, when diabetic podocytes were treated with 20 μM tangeretin. On the other hand, the dihydroethidium (DHE) staining showed that the ROS generation was enhanced in diabetic glomeruli (Figure 5B). However, oral administration of 10 mg/kg tangeretin reduced the glomerular ROS production in db/db mice.

Aquaporins are integral membrane proteins involved in water transport across cell membranes and especially aquaporin 1 (AQP1) is located in glomerular podocytes, in addition to proximal convoluted tubule epithelium [33]. In diabetic podocytes the expression of AQP1 was enhanced during the oxidant generation (Figure 5C). In contrast, tangeretin reduced its induction in a dose-dependent manner. On the other hand, the expression of antioxidant enzyme superoxide dismutase 2 (SOD2) was down-regulated in podocytes treated with 33 mM glucose (Figure 5C). When glucose-loaded the podocytes were treated with 1–20 μM tangeretin for 4 day, the SOD2 induction was dose-dependently prompted.

### 2.6. Suppressive Effects of Tangeretin on Hypoxia-Induced Podocyte Injury

**Figure 6:**
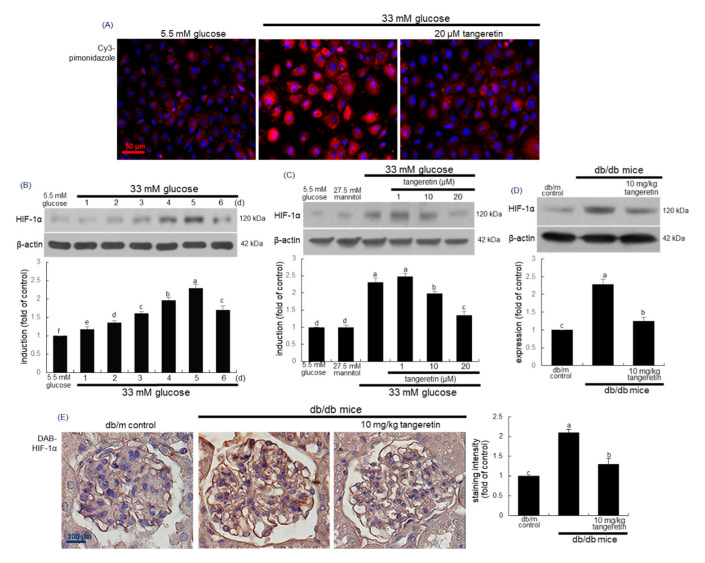
Pimonidazole induction (**A**), temporal expression of HIF-1α (**B**) and inhibition of HIF-1α expressions by tangeretin in 33 mM glucose-exposed podocytes (**C**) and diabetic kidney (**D**,**E**). Podocytes were incubated in media containing 5.5 mM glucose, 5.5 mM glucose plus 27.5 mM mannitol as osmotic controls or 33 mM glucose in the absence and presence of 1–20 μM tangeretin for 4 days. For the immunocytochemical analysis of pimonidazole (**A**), a Cy3-conjugated secondary antibody was used to visualize the induction of pimonidazole. The nuclear staining was done with DAPI (blue). Each photograph is representative of stained cells. Magnification: 400-fold. The db/db mice were orally supplemented with 10 mg/kg tangeretin for 10 weeks. Cell lysates and tissue extracts were electrophoresed on 8–10% SDS-PAGE and subject to Western blot analysis with a primary antibody against HIF-1α (**A**–**C**). β-Actin antibody was used as an internal control. The bar graphs (mean ± SEM, *n* = 3) represent quantitative results of blot bands obtained from a densitometer. Values in bar graphs not sharing a common letter are significantly different at *p* < 0.05. Immunohistochemical staining was conducted to reveal tissue levels of HIF-1α of diabetic glomeruli (**E**). The glomerular HIF-1α was confirmed with 3,3′-diaminobenzidine (DAB), producing a brown staining, counterstained with hematoxylin and was quantified. Each photograph is representative of four mice. Scale bar = 50 μm or 200 μm.

To investigate oxygen tension within podocytes, this study employed pimonidazole staining widely used to detect cellular and tissue hypoxia. Immunocytochemical analysis revealed that the Cy3-red fluorescent staining of pimonidazole strongly increased in 33 mM glucose-exposed podocytes (Figure 6A). When diabetic podocytes were incubated in the presence of 20 μM tangeretin for 4 days, the pimonidazole induction declined to the glucose control level (Figure 6A).

The knockdown of SOD2-dependent ROS generation up-regulates HIF-1α expression in carcinoma cells [34,35]. When podocytes were exposed to glucose for up to 6 days, the cellular expression level of HIF-1α increased in a temporal manner, indicative of hyperglycemia-induced podocyte hypoxia (Figure 6B). However, 1–20 μM tangeretin reduced the HIF-1α expression in glucose-loaded podocytes (Figure 6C). Consistently, the tissue level of HIF-1α was elevated in diabetic mouse kidneys (Figure 6D). When 10 mg/kg tangeretin was orally administrated to db/db animals for 10 weeks, the HIF-1α induction was diminished in diabetic kidneys. Further, the immunohistochemical staining of HIF-1α supported the Western blot data showing that tangeretin curtailed the tissue induction of HIF-1α in diabetic mouse kidneys (Figure 6E).

### 2.7. Inhibitory Effects of Tangeretin on Hypoxia and EMT Induced by CoCl_2_

**Figure 7:**
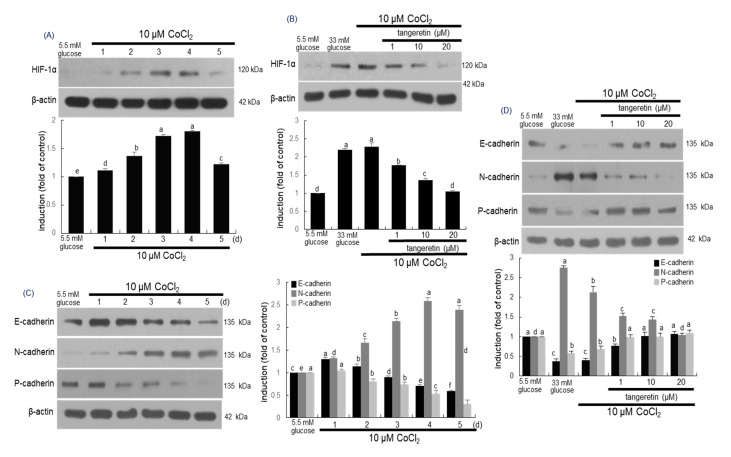
Temporal expression of HIF-1α (**A**), E-cadherin, N-cadherin and P-cadherin (**C**) and their inhibition by tangeretin (**B**,**D**) in CoCl_2_–stimulated podocytes. Podocytes were incubated with 10 μM CoCl_2_ in the presence of 1–20 μM tangeretin for up to 5 days. Podocyte lysates were electrophoresed on 8–10% SDS-PAGE and subject to Western blot analysis with a primary antibody of HIF-1α, E-cadherin, N-cadherin and P-cadherin. β-Actin antibody was used as an internal control. The bar graphs (mean ± SEM, *n* = 3) represent quantitative results of each blot bands obtained from a densitometer. Values in bar graphs not sharing a common letter are significantly different at *p* < 0.05.

It has been reported that CoCl_2_ artificially induce the hypoxia by blocking the degradation of HIF-1α that stimulates transcription of hypoxia associated genes [36]. As expected, cellular expression of HIF-1α was enhanced in podocytes exposed 10 μM CoCl_2_ for up to 5 days (Figure 7A). The induction of HIF-1α by CoCl_2_ was similar to its induction by stimulation with 33 mM glucose (Figure 6B). Submicromolar tangeretin reduced the HIF-1α expression of CoCl_2_-exposed podocytes in a dose-dependent manner (Figure 7B).

This study attempted to examine whether hypoxia played a crucial role in inducing EMT via activating HIF-1α. Several studies have shown that hypoxia-induced oxidative stress might be involved in cell damage through the stimulation of angiogenesis and apoptosis [37,38]. When podocytes were exposed to 10 μM CoCl_2_ for up to 5 days, the cellular levels of epithelial markers of E-cadherin and P-cadherin reduced in a temporal manner (Figure 7C). In contrast, the expression of the mesenchymal N-cadherin was consistently enhanced (Figure 7C). On the other hand, the induction of E-cadherin, P-cadherin and N-cadherin was reciprocally reversed by treating the hypoxic podocytes with 1–20 μM tangeretin (Figure 7D).

**Figure 8:**
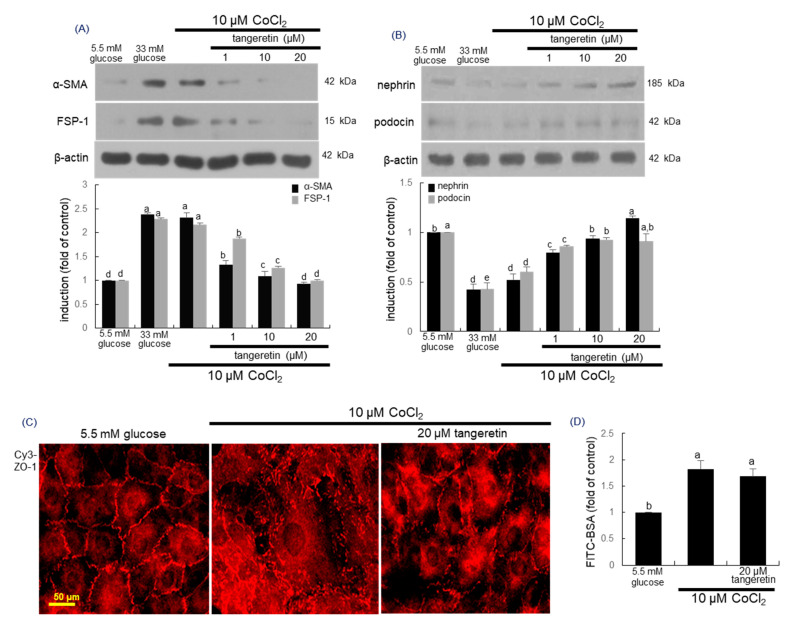
Effects of tangeretin on expression of α-SMA, FSP-1, nephrin, podocin and ZO-1 in CoCl_2_ -exposed podocytes (**A**,**B**,**D**). Podocytes were incubated with 10 μM CoCl_2_ in the presence of 1–20 μM tangeretin for 4 days. Podocyte lysates were electrophoresed on 8–10% SDS-PAGE and subject to Western blot analysis with a primary antibody of α-SMA, FSP-1, nephrin and podocin. β-Actin antibody was used as an internal control. The bar graphs (mean ± SEM, *n* = 3) represent quantitative results of each blot bands obtained from a densitometer. The ZO-1 localization was visualized with a Cy3-conjugated secondary antibody (**C**). Each photograph is representative of stained cells. Scale bar = 50 μm. For the measurement of podocyte injury, the albumin permeability assay was performed with FITC-labeled bovine serum albumin (BSA), (**D**). Means in bar graphs without a common letter differ, *p* < 0.05.

This study further investigated that tangeretin inhibited hypoxia-induced transdifferentiation of podocytes into myofibroblasts. Cellular expression levels of the myofibroblast markers of FSP-1 and α-SMA were markedly elevated by 10 μM CoCl_2_ (Figure 8A). Thus, experimental hypoxia may promote phenotypic alterations and acquisition of mobility. However, such elevation was highly blocked by treating 1–20 μM tangeretin (Figure 8A).

### 2.8. Inhibition of Hypoxia-Induced Disruption of Adherens Junction by Tangeretin

The slit diaphragm represents an adherens junction composed of P-cadherin catenin proteins and ZO-1 [39]. This study investigated that hypoxia influenced the expression of the slit diaphragm proteins and ZO-1 in podocytes, which was beneficially ameliorated by treating tangeretin to those cells. The expression levels of nephrin and podocin were significantly diminished by the stimulation with 10 μM CoCl_2_ (Figure 8B). However, 1–20 μM tangeretin supplemented to hypoxic cells dose-dependently enhanced the expression of these proteins. Further, the immunocytochemical data showed that 10 μM CoCl_2_ disrupted ZO-1-based junction of podocytes (Figure 8C). In contrast, the treatment of CoCl_2_-exposed podocytes with 20 μM tangeretin enhanced the reduced expression of ZO-1 for maintaining the unique cell junction. To confirm that hypoxia induced podocyte foot process effacement due to disruption of cellular junctions, FITC-conjugated albumin permeability assay was carried out. When podocytes were exposed to 10 μM CoCl_2_, the permeability of the FITC-labeled bovine serum albumin (BSA) was augmented (Figure 8D). On the contrary, the treatment of 20 μM tangeretin did not curtail such permeability, indicating that the presence of tangeretin cannot repair the chemical damage of cellular junctions regardless of the up-regulation of junction proteins.

## 3. Discussion

The visceral epithelial podocytes play an important role in supporting glomerular structure and function and forming a filtration barrier [3,4]. These cells collaborate elaborately with mesangial cells, endothelial cells of the glomerular capillary loop and the glomerular basement membrane [2,3]. The slit diaphragm is a zipper-like structure and a specialized type of intercellular junction that connects neighboring podocyte foot processes [5]. When podocytes become injured by diverse stimuli including glucose and ROS, they undergo foot process effacement and loss of slit diaphragms, leading to the development of proteinuria in podocytopathies [6,7]. EMT is a process by which podocytes lose their epithelial characteristics and acquire migratory and invasive properties of mesenchymal cells [8,9]. This process contributes to lose its phenotype and disintegrate their junction ability [8]. It is widely recognized that EMT is a key process that contributes to diabetic nephropathy (DN), resulting hyperglycemia-mediated kidney fibrosis and proteinuria [9,10]. This study showed that glucose induced the EMT in in podocytes and diabetic kidneys podocytes by loss of E-cadherin and P-cadherin, acquisition of mesenchymal N-cadherin and formation of myofibroblasts [10]. Accordingly, podocyte depletion has been proposed as a strategy for the establishment of a novel therapy for proteinuria was proposed [9,11].

Numerous studies with naturally-occurring polyphenols have shown that a direct blockade targeting podocyte EMT ameliorates podocyte injury and proteinuria [23,40]. In addition, podocyte slit diaphragm has been a therapeutic target of proteinuria [5,41]. This study found that the polymethoxylated flavone tangeretin inhibited EMT process in diabetic podocytes and diabetic mouse kidneys. As a result, tangeretin ameliorate slit diaphragm function leading to the reduction of protein excretion in urine. A study has reviewed the molecular mechanisms involved in podocyte EMT and its related diabetic kidney diseases [10]. Hyperglycemia evokes podocyte EMT through several molecular mechanisms including TGF-β/Smad pathway, Wnt/β-catenin signaling pathway and integrins/integrin-linked kinase signaling pathway [42,43,44]. A recent paper shows that the citrus flavanone hesperetin blocks EMT in TGF-β-treated podocytes via regulation of mTOR signaling [45]. Thus, one can assume that tangeretin inhibited TGF-β-mediated EMT in glucose-exposed podocytes. In addition, reduced autophagy induces EMT and contributes to fibrosis via aberrant epithelial–fibroblast crosstalk [46]. Plant-derived saponin astragaloside IV attenuates glucose-induced podocyte EMT through enhancing autophagy [47]. Similarly, it can be speculated that tangeretin may modulate crosstalk between glucose and autophagy and block fibrotic injury of diabetic podocytes. In this study oxidative stress was involved in glucose-induced podocyte injury caused by elevated EMT. Tangeretin inhibited diabetic injury of podocytes through diminishing oxidative stress-induced DNA damage in the form of 8-OHdG and reducing the induction of AQP1 that might facilitate ROS diffusion into podocytes [48]. In diabetic podocytes tangeretin improved mitochondrial antioxidant defense by enhancing SOD2 induction. Mitochondrial oxidative stress and biological regulation of SOD2 plays a role in the pathogenesis of kidney diseases [49]. In addition, dietary tangeretin inhibited apparent production of ROS observed in diabetic glomeruli with foot process effacement and loss of cell-cell junction proteins.

Both hyperglycemia and hypoxia have been suggested to be major factors for diabetic complications [50,51]. The development of kidney hypoxia has been accepted as a common pathway to DN. Hyperglycemia increases HIF-1α accumulation and disrupts HIF-1α stability that causes cellular damage in diabetes via several mechanisms [51]. Tangerine effectively inhibited HIF-1α induction in glucose-loaded podocyte and diabetic kidneys. Accordingly, one can assume that hyperglycemia-induce hypoxia may cause podocyte injury through accelerating EMT process and myofibroblast formation of diabetic podocytes. This study introduced hypoxia-mimetic experiments with CoCl_2_ in order to explore the crosstalk among hypoxia, EMT and disruption of cell-cell junction. The artificial hypoxia inducer CoCl_2_ affected the induction of EMT-related proteins and slit diaphragm proteins. Tangeretin attenuated the concomitant induction of these proteins with increased level of HIF-1α in CoCl_2_-loaded podocytes. It should be noted that the treatment of tangeretin did not repair the chemical damage of cellular junctions regardless of the up-regulation of junction proteins. On the other hand, the interplay between ROS and hypoxia is complex, bidirectional and still not completely explained. As stated above, hyperglycemia induced both oxidative stress and cellular hypoxia in diabetic podocytes and diabetic kidneys. [49,51]. Mitochondrial function in the diabetic kidney appeared to be intertwined in a complex manner, being connected to mitochondrial ROS production and the development of kidney hypoxia in DN [52].

In summary, this study investigated that tangeretin inhibited podocyte injury and fibrosis through blocking EMT due to oxidative stress and hypoxia caused by hyperglycemia. Tangeretin inhibited loss of epithelial phenotypes, mesenchymal transition and myofibroblast-like cell formation in diabetic podocytes. In addition, tangeretin attenuated loss of slit diaphragm proteins and adherenes junction protein of ZO-1, leading to filtration slit damage and podocyte foot process effacement. Oral treatment of tangeretin to animals counteracted loss and foot process effacement of podocytes during EMT process of diabetic mouse kidneys. Further, tangeretin blocked the glucose- and CoCl_2_-induced HIF-1α expression and ROS generation in podocytes and glomeruli. Therefore, tangeretin may be a potent agent inhibiting podocyte injury and fibrosis through blocking podocyte EMT caused by glucose-induced oxidative stress and hypoxia.

## 4. Materials and Methods

### 4.1. Materials

RPMI-1640 media, mannitol, D-glucose and tangeretin were obtained from Sigma-Aldrich Chemical (St Louis, MO, USA), as were all other reagents, unless specifically stated elsewhere. Fetal bovine serum (FBS), trypsin-ethylenediaminetetraacetic acid and penicillin-streptomycin were purchased from Lonza (Walkersvillle, MD, USA). Rabbit polyclonal antibodies of FSP-1, HIF-1α and N-cadherin were obtained from Abcam Biochemicals (Cambridge, UK). Mouse monoclonal antibodies of α-SMA, E-cadherin, P-cadherin, ZO-1, nephrin, 8-OHdG), AQP1 and SOD2 were supplied by Santa Cruz Biotechnology (Santa Cruz, CA, USA). Rabbit polyclonal podocin antibody and mouse monoclonal β-actin antibody were provided by Sigma-Aldrich Chemical. Horseradish peroxidase (HRP)-conjugated goat anti-rabbit IgG, goat anti-mouse and donkey anti-goat IgG were purchased from Jackson ImmumnoReserch Laboratories (West Grove, PA, USA). 4′,6-Diamidino-2-phenylindole (DAPI) was obtained from Santa Cruz Biotechnology.

Tangeretin was dissolved in dimethyl sulfoxide (DMSO) for live culture with cells; a final culture concentration of DMSO was <0.5%.

### 4.2. Murine Kidney Podocyte Culture

Conditionally immortalized mouse podocytes were purchased from the Cell Line Service (Eppelheim, Germany). Cells were cultured in RPMI-1640 culture media containing 10% FBS, 100 U/mL penicillin and 100 μg/mL streptomycin at 37 °C humidified atmosphere of 5% CO_2_ in air. Podocytes were sub-cultured at 90% confluence for further experiments. To induce a hyperglycemic condition, podocytes were incubated in media containing 33 mM glucose, in comparison with normal media containing 5.5 mM glucose. Cells were further treated with 1–20 μM tangeretin for 4 days. Cells were also incubated in media containing 5.5 mM glucose and 27.5 mM mannitol as an osmotic control. In another set of experiments, podocytes were exposed to 10 μM CoCl_2_ for the induction of hypoxic condition [53]. Cells were treated with 10 μM CoCl2 in the absence and presence of 1–20 μM tangeretin for 4 days.

After podocytes were exposed to 33 mM glucose, 10 μM CoCl_2_ and/or 1–20 μM tangeretin, 3-(4,5-dimetylthiazol-yl)-diphenyl tetrazolium bromide (MTT, Duchefa Biochemie, Haarlem, Netherlands) assay was conducted in order to quantitate cellular viability. Podocyte viability was determined using a colorimetric assay based on the uptake of MTT by viable cells. When podocytes were treated with 33 mM glucose for up to 5 days in the absence and presence of 1–20 μM tangeretin, no alteration in cell viability was detected (Figure 1B).

### 4.3. Western Blot Analysis

Western blot analysis was conducted using whole cell lysates prepared from podocytes (3.5 × 10^5^ cells/well plate) and renal tissue extracts of mice. Whole cell lysates and renal cortical tissues were prepared in a lysis buffer containing 1 M β-glycerophosphate, 1% β-mercaptoethanol, 0.5 M NaF, 0.1 M Na_3_VO_4_ and protease inhibitor cocktail. Cell lysates and renal tissue extracts containing equal amounts of proteins were electrophoresed on 6–15% SDS-PAGE and transferred onto a nitrocellulose membrane. Nonspecific binding was blocked with 5% skim milk for 3 h. The membrane was incubated overnight at 4 °C with each primary antibody of target proteins and washed in a Tris buffered saline-Tween 20 for 10 min each three times. The membrane was then incubated for 1 h with a secondary antibody of goat anti-rabbit IgG, goat anti-mouse IgG and rabbit anti-goat IgG conjugated to horseradish peroxidase (HRP). Each target protein level was determined by using immobilon western chemiluminescent HRP substrate (Millipore Corporation, Billerica, MA, USA) and Agfa X-ray film (Agfa, Gevaert, Belgium). Incubation with mouse monoclonal β-actin antibody was also performed for comparative controls.

### 4.4. In Vivo Mouse Experiments

The current study employed adult male db/db mice (C57BLKS/+Leprdb Iar; Jackson Laboratory, Sacramento, CA, USA) and their age-matched non-diabetic db/m littermates (C57BLKS/J; Jackson Laboratory). Mice were kept on a 12 h light/12 h dark cycle at 23 ± 1 °C with 50 ± 10% relative humidity under specific pathogen-free conditions, fed a standard pellet laboratory chow diet (Cargill Agri Purina, Biopia, Korea) and were supplied by the animal facility of Hallym University. This study used animals at 7 weeks of age because they begin to develop diabetes (hyperglycemia) at the age of 7–8 weeks. The animals were allowed to acclimatize for a week before beginning the feeding experiments. Mice were divided into three subgroups (*n* = 12 for each subgroup). Mice of the first group were non-diabetic db/m control mice and db/db mice were divided into two subgroups. One group of db/db mice was orally administrated 10 mg/kg tangeretin daily for 10 weeks.

All animal experiments were approved by the Committee on Animal Experimentation of Hallym University and performed in compliance with the University’s Guidelines for the Care and Use of Laboratory Animals (hallym 2017–35).

### 4.5. Immunohistochemical Staining

For the immunohistochemical analysis, paraffin-embedded mouse kidney tissue sections (5 μm thick) were employed. The sections were placed on glass slides, de-paraffinized and hydrated with xylene and graded alcohol. The sections were pre-incubated in a boiled sodium citrate buffer (10 mM sodium citrate, 0.05% Tween 20, pH = 6.0) for antigen retrieval. Each specific primary antibody against α-SMA, nephrin or HIF-1α was incubated with the tissue sections overnight. For the measurement of α-SMA expression, the tissue section was stained with FITC-conjugated anti mouse IgG. In addition, nephrin and HIF-1α were visualized with 3,3′-diaminobenzidine (DAB) to produce a brown staining, being counterstained with hematoxylin. The stained tissue sections were examined using an optical Axioimager microscope system and five images were taken for each section (Zeiss, Oberkochen, Germany).

### 4.6. Urinary Albumin Excretion

In animal experiments the 24-h urine collection was carried out by using metabolic cages. Urinary albumin excretion was examined by using an enzyme-linked immunosorbent assay (ELISA) kit (Abcam Biochemicals), according to the manufacturer’s instruction.

### 4.7. TEM Analysis

Freshly prepared kidney tissues of mice were immediately fixed for 3 days in 2.5% glutaraldehyde in 0.1 M Sorenson’s phosphate buffer (pH = 7.4) and post-fixed in 1% OsO_4_, followed by washing in distilled water and en bloc staining in 3% uranyl acetate. Dehydration was conducted using a 70–100% graded ethanol. The tissues were then embedded in Epon polymer and ultra-thin sections (70 nm) were obtained with a diamond knife on a Reichert Jung ultramicrotome, stained with uranyl acetate-lead citrate and observed with a Philips CM-100 transmission electron microscope (FEI instruments, Hillsborough, OR, USA) at an accelerating voltage of 60 kV. The TEM images with magnifications ranging from 130,000× were analyzed for the examination of the morphology of subcellular constituents and structures.

### 4.8. Immunocytochemical Staining

Immunofluorescent cytochemical staining was performed to examine the induction of ZO-1 and 8-OHdG in podocytes. The podocytes were fixed with 4% formaldehyde for 10 min and permeated with 0.1% Triton X-100 for 10 min on ice. The cells were blocked with 20% goat serum for 1 h and then incubated with a primary antibody against ZO-1 or 8-OHdG and a secondary antibody of Cy3-conjugated IgG or FITC-conjugated IgG. In another set of experiments for the detection of intracellular hypoxia, podocytes were cultured in fresh RPMI-1640 containing 10 μM pimonidazole hydrochloride (Hypoxyprobe, Burlington, MA, USA) for 1 h. Subsequently, immunocytochemical staining was performed with a primary antibody against pimonidazole (clone 4.3.11.3) and a secondary antibody of Cy3-conjugated IgG for 30 min. Nuclear staining was performed with 4′,6-diamidino-2-phenylindole (DAPI). Each slide was mounted in a mounting medium and images from each slide were taken using an optical Axioimager microscope system.

### 4.9. Albumin Permeability Assay

The albumin permeability assay was conducted using transwell inserts with 8 μm pore size filters (Costar, Corning, NY, USA). Podocytes (3.0 × 10^4^ cells/24-well plate well) were added onto insert chamber and treated with 5.5 mM glucose or 33 mM glucose in absence and presence of 20 μM tangeretin at 37 °C for 4 days. After changing to serum-free media, the BSA-labeled with 50 μg/mL FITC was treated onto the upper chamber. After 1 h diffusion, media in lower chamber compartment was collected and measured fluorescence intensity using Fluoroskan reader (Thermo Fisher Scientific, Waltham, MA, USA) at respective emission and excitation wavelengths of 495 and 520 nm.

### 4.10. Measurement of ROS Production

Paraffin-embedded tissue sections (5 μm thickness) of kidneys were deparaffinized and hydrated for the dihydroethidium (DHE) staining. Kidney tissues were stained by incubating with 20 μM DHE (Invitrogen, Carlsbad, CA, USA) for 1 h. For the identification of nuclei, DAPI was treated for 10 min. Stained tissues on slides were mounted in mounting solution and images of each slide were taken using an optical microscope Axioimager system.

### 4.11. Data Analysis

The results are presented as mean ± SEM. Statistical analyses were conducted using the Statistical Analysis Software package, version 6.12 (SAS Institute, Cary, NC, USA). Significance was determined by one-way ANOVA, followed by Duncan’s multiple-range test for multiple comparisons. Differences were considered significant at *p* < 0.05.

## Abbreviations

AQP1	aquaporin-1
CoCl_2_	cobalt chloride
DN	diabetic nephropathy
EMT	epithelial-to-mesenchymal transition
FSP-1	fibroblast-specific protein-1
HIF-1α	hypoxia inducible factor-1α
8-OHdG	8-hydroxy-2-deoxy guanosine
α-SMA	α-smooth muscle actin
SOD2	superoxide dismutase 2
ZO-1	zonula occludens-1
